# Kinematic Determination of the Aerial Phase in Ski Jumping

**DOI:** 10.3390/s22020540

**Published:** 2022-01-11

**Authors:** Ola Elfmark, Gertjan Ettema, Petter Jølstad, Matthias Gilgien

**Affiliations:** 1 Department of Civil and Environmental Engineering, Norwegian University of Science and Technology, 7491 Trondheim, Norway; 2 Norwegian Olympic and Paralympic Committee and Confederation of Sports, 0863 Oslo, Norway; 3 Department of Neuromedicine and Movement Science, Norwegian University of Science and Technology, 7491 Trondheim, Norway; gertjan.ettema@ntnu.no; 4 Department of Physical Performance, Norwegian School of Sport Sciences, 0863 Oslo, Norway; petter.jolstad@gmail.com (P.J.); matthias.gilgien@nih.no (M.G.); 5 Center of Alpine Sports Biomechanics, Engadin Health and Innovation Foundation, 7503 Samedan, Switzerland

**Keywords:** ski jumping, dGNSS, LD-ratio, stable flight, steady glide

## Abstract

The purpose of this study was to find a generic method to determine the aerial phase of ski jumping in which the athlete is in a steady gliding condition, commonly known as the ‘stable flight’ phase. The aerial phase of ski jumping was investigated from a physical point mass, rather than an athlete–action-centered perspective. An extensive data collection using a differential Global Navigation Satellite System (dGNSS) was carried out in four different hill sizes. A total of 93 jumps performed by 19 athletes of performance level, ranging from junior to World Cup, were measured. Based on our analysis, we propose a generic algorithm that identifies the stable flight based on steady glide aerodynamic conditions, independent of hill size and the performance level of the athletes. The steady gliding is defined as the condition in which the rate-of-change in the lift-to-drag-ratio (LD-ratio) varies within a narrow band-width described by a threshold τ. For this study using dGNSS, τ amounted to 0.01 s^−1^, regardless of hill size and performance level. While the absolute value of τ may vary when measuring with other sensors, we argue that the methodology and algorithm proposed to find the start and end of a steady glide (stable flight) could be used in future studies as a generic definition and help clarify the communication of results and enable more precise comparisons between studies.

## 1. Introduction

**Figure 1:**
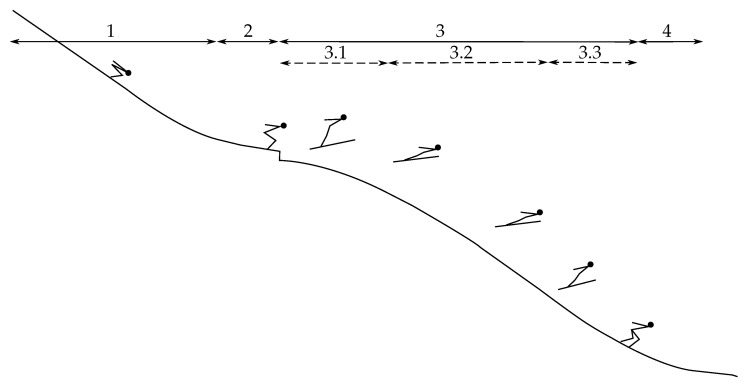
Graphical overview of a ski jump with the separate phases a ski jump is divided into. 1: Inrun, 2: take-off, 3: flight, 3.1: early flight, 3.2: Stable flight (steady glide), 3.3: landing preparation, 4: landing.

Ski jumping is a popular winter sport, with an Olympic history dating back to 1924 [1]. In previous research, the consensus has been to divide a ski jump into four main phases: inrun, take-off, flight, and landing, where the flight phase is subdivided into three phases: early flight, stable flight, and landing preparation [2], as shown in Figure 1. A ski jumper aims to gain the highest speed possible through the inrun [3], while establishing the best possible conditions for the phase considered to be most influential on performance, the take-off, where the initial conditions for the flight are created [2,4,5]. The early flight phase spans from where the ski jumper is airborne until a steady flight posture is reached. The aim of the early flight phase is to reach stable flight as quickly as possible, with minimal speed loss [6,7,8,9].

The stable flight phase is the part where the ski jumper maintains a fairly constant flight posture, i.e., from the end of early flight to the start of landing preparation. In this phase, the aerodynamic forces are highly influential and the ski jumper intends to achieve as high of a lift-to-drag-ratio (LD-ratio) as possible [2]. From a physics point of view, a ski jumper must produce a force, counteracting the gravitational force in order to be flying. However the aerodynamic forces do not counteract the gravitational forces, and the ski jumper does not provide any propulsion or thrust. Therefore, a more accurate description would be ‘gliding’, as the ski jumper glides through the air, and descends gradually without supplying force. Performance is to a great extent determined by the initial conditions determined by the take-off and the gravitational and aerodynamic forces through the glide phase [10,11,12]. The term ‘stable’ is probably derived from the limb motions of the ski jumper, which are relatively static during this phase compared to the take-off and landing phases. The dynamics of the athlete is not as tightly coupled to the flight characteristics as the effect of the athlete’s action is. Therefore, this phase should be described as ‘isometric-static’ or ‘steady’, i.e., the rate-of-change of aerodynamic forces is close to zero. Hence, the term ’steady glide’ will be used for the ’stable flight’ phase (3.2 in Figure 1) through this article.

While much research has examined factors influencing take-off and early flight, research on the steady glide phase is scarce. This was partly caused by the difficulties in establishing valid methods to collect data from this phase, since the ski jumper moves through a large volume at high speed. Currently, researchers have used computational fluid dynamic simulations and wind tunnel measurements, and there have been a small number of studies using video analysis to examine how the glide posture can influence glide parameters, such as the aerodynamic relationship between the lift ($F_{L}$) and drag forces ($F_{D}$), i.e., LD-ratio [9,13,14,15,16,17,18,19]. In recent years, wearable sensors as inertial measurement units (IMUs) have shown promising results when measuring kinematics and kinetics from the start of the inrun to the landing [20,21,22,23,24,25,26,27,28]. A suitable method used to provide accurate field data from the steady glide phase is differential global navigation satellite system (dGNSS) technology. Measurements with dGNSS are extensively used and validated in alpine skiing for position, velocity, and acceleration-related parameters [29,30,31,32,33], but have rarely been used in ski jumping [34]. However, a recent study has shown the possibilities dGNSS provides to efficiently collect data from the start of the inrun to the landing [35]. The use of this method allows measurement of the trajectory with ± 0.05 m global position accuracy [36]. As long as carrier phase kinematic double difference ambiguities can be fixed, the position accuracy of geodetic receivers and high antenna quality is such that velocity and acceleration, derived from position-time information, can be used to describe the aerodynamic forces during the glide [35].

To investigate the aerial phase from a physical, rather than an athlete-action-centered perspective, reliable definitions of the phases are required. The start of the early flight and the end of the landing preparation phase are defined as being when the athlete leaves and reengages with the ground. The start and end of the steady glide phase is more challenging, but still require a functional delimitation to the glide and landing preparation phase. Consequently, the aim of this study was to proposea definition to determine the beginning and end of the steady glide phase, where the steady glide phase is defined as the part of the aerial phase where the rate-of-change in the LD-ratio is close to zero. For that purpose, dGNSS was used to collect data from four different hill sizes, with ski jumpers of various performance levels to cover a range of conditions and athletes, in order to develop a robust and widely applicable definition of the steady glide phase.

## 2. Materials and Methods

Four separate data collections were carried out to capture the range among small, intermediate, and large hill sizes. Information about the data collections is provided in Table 1.

HS77 is a small hill used by juniors and in training sessions. HS106 and HS117 are referred to as ‘normal hills’ and HS140 as a ‘large hill’ in ski jumping, and are representative of the two hill sizes used in World Cup and Olympic competitions. The steady glide phase was investigated across hill sizes and performance levels, due to the span of the data sets. The study was conducted in accordance with the Declaration of Helsinki [37], approved by the Norwegian Centre for Research Data and the ethical committee of the Norwegian School of Sport Sciences.

### 2.1. dGNSS Measurement

**Figure 2:**
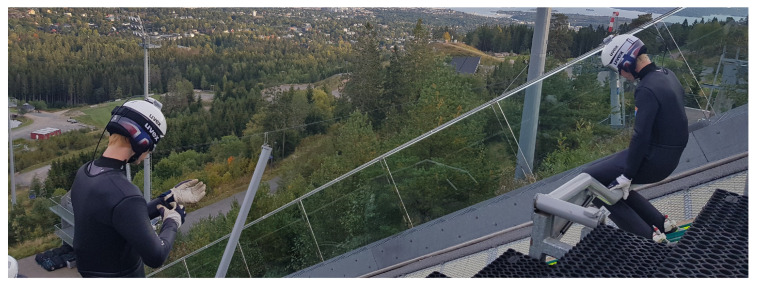
Ski jumpers with the dGNSS antenna mounted on their helmets and the receivers in backpacks that were carried under the ski jumping suit. Picture from [35].

In each data collection, the same dGNSS method was applied, in which the head trajectories of the athletes were captured using a dGNSS with a receiver (Alpha-G3T, Javad, CA, USA) in a backpack and an antenna (G5Ant-2AT1, Antcom, Torrance, CA, USA) mounted on the helmet, as shown in Figure 2. The antenna mounting point can be considered a reasonable representation of the athlete as a point mass as soon as the ski jumper has reached a steady posture, but not during the take-off and landing phases [35].

A GNSS base station with antenna (GrAnt-G3T, Javad, San Jose, CA, USA) and receiver (Alpha-G3T, Javad, San Jose, CA, USA) was positioned in close proximity to the inrun (short-baseline dGNSS). GNSS signal reception through the GNSS antenna requires a direct line of sight to the satellites and is therefore constrained to being mounted on the head of the ski jumper. Both the athlete’s GNSS and the base station GNSS logged GPS/GLONASS dual frequency (L1/L2) signals at 50 Hz. Raw GNSS data were downloaded from the GNSS receivers and dGNSS solutions were calculated in post-processing. Geodetic dGNSS post-processing was done using the software Justin (Javad, San Jose, CA, USA) and was applied to the raw GNSS data to calculate kinematic carrier phase double difference position solutions [36], which were exported in a Cartesian coordinate system (UTM32N), and rotated in to a local coordinate system with the origin at the inrun edge with *x* along the horizontal axis of the jump and *y* along the vertical axis. For the analysis, jumps were only used when the dGNSS solution managed to fix the integer ambiguities in the dGNSS solution yielding a typical global position error of ± 0.05 m. Jumps with periods of dGNSS float solutions (typically yielding one order of magnitude larger errors [36]) were discarded from the analysis.

The raw dGNSS positions were filtered with a weighted cubic spline filter, where position error estimates from the geodetic dGNSS processing were applied as weights [38,39]. Position, velocity, and acceleration were derived from the dGNSS position—time data. The LD-ratio was calculated as a coefficient from the horizontal and vertical acceleration components. For more detailed information about the calculation of parameters, the reader is referred to Elfmark et al. [35]. The derived position, velocity, and acceleration data were filtered with different cut-off frequency settings using a second order Butterworth filter, as stated in Appendix A. These were used to test the sensitivity of filter settings for the identification of the steady glide phase as described in Section 3. Parameter calculation, filtering, and the following analysis were conducted in Matlab R2021a.

### 2.2. The Rationale behind the Determination of the Steady Glide Phase

For a jump to be successful, we hypothesize that a steady glide is obtained during a certain time period and distance. A steady glide phase entails a period in which changes in drag and lift forces and, hence, the LD-ratio, are small, but exist to a certain extent, while the variability in the LD-ratio is substantially larger prior to and following the steady glide phase. The change in LD-ratio variability at the start and end of the steady glide phase is deployed in this study to identify this phase. Hence, the main challenge in identifying the steady glide phase is the setting of LD-ratio variation boundaries to successfully divide the ‘steady’ from the non-steady phases. An algorithm was built to search for the start and end of the steady glide phase. The search started in the mid-section of where the steady glide phase was assumed. For that purpose, the starting position of the search (p${}_{m}$) was set at 40 m after the take-off for all hill sizes included in this study. Then, the algorithm searched the LD-ratio rate-of-change signal towards the take-off; i.e., backward in time. The start of the steady glide was defined as the first point between p${}_{m}$ and 0 m (inrun edge), where the rate-of-change exceeded a set band-width described by a threshold (τ) and the end point was defined as the first point exceeding τ after p${}_{m}$. The steady glide phase (distance) was hence defined as the period in which the LD-ratio was continuously within that τ-range, both backward (toward take-off, 0 m) and forward (toward the landing zone) from p${}_{m}$.

Furthermore, we assumed the steady glide period to be reasonably consistent, both in terms of distance and actual LD-ratio, between jumps and jumpers. The rate of change in the LD-ratio at p${}_{m}$ is (by definition) close to zero in all jumps, and its (inter-jump) standard deviation in LD-ratio is used as the initial threshold value ($\tau_{0}$) for the the LD-ratio range. This threshold was used as the initial input parameter in the algorithm that searched for the minimization of four parameters: the standard deviation of the start and end positions of the steady glide phase (SD${}_{1,2}$) and the standard deviation of the LD-ratios at these points (SD${}_{3,4}$). The obtained standard deviations over a range of τ-values were normalized with their own mean as

(1) $${\mathrm{nSD}}_{1-4.\tau }=\frac{{\mathrm{SD}}_{1-4.\tau }}{\bar{{\mathrm{SD}}_{1-4}}}.$$

The mean value of the four nSD${}_{1-4.\tau }$ from (1) was the cost function ($\bar{{\mathrm{nSD}}_{1-4.\tau }}$), and the optimal τ ($\tau_{opt}$) was defined as at the τ where the absolute value of the cost function was minimal.

## 3. Results and Discussion

The first part of this analysis highlights how the $\tau_{opt}$ was determined from the analysis of SD${}_{1-4}$ and the cost function. For that purpose, the filter settings for the parameters were set to the same values as in Elfmark et al. [35]. In the second part, the effect of the filter settings is addressed, since these can be highly influential on this type of data. To develop the algorithm for these two first parts, the data from HS106 was chosen, since this data collection contained the largest number of jumps. Once the algorithm was developed, it was applied with the chosen filter settings on all four data sets (HS140, HS106, HS117, and HS77) to investigate whether the same algorithm and filter settings would be able to identify the steady glide phase for different hill sizes and performance levels of the ski jumpers.

### 3.1. Determination of $\tau_{opt}$

**Figure 3:**
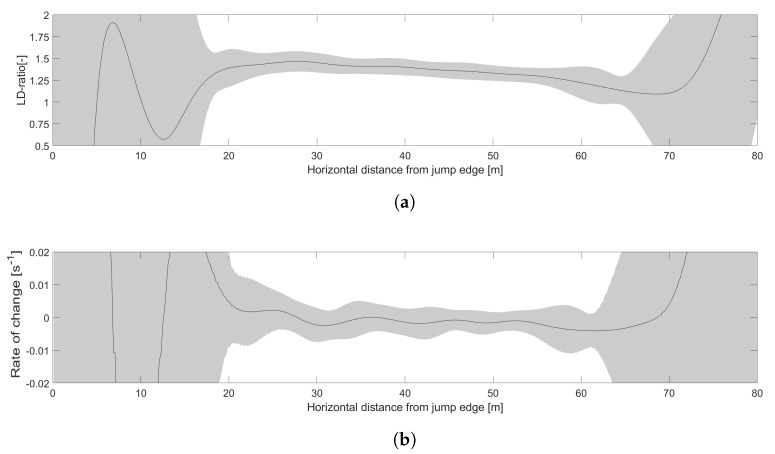
Average values of LD-ratio, shown in (**a**) and rate-of-change of the LD-ratio in (**b**) with *n* = 38. Gray shaded error bands show the standard deviation.

Average values of LD-ratios and rate-of-change for HS106 are shown in Figure 3.

**Figure 4:**
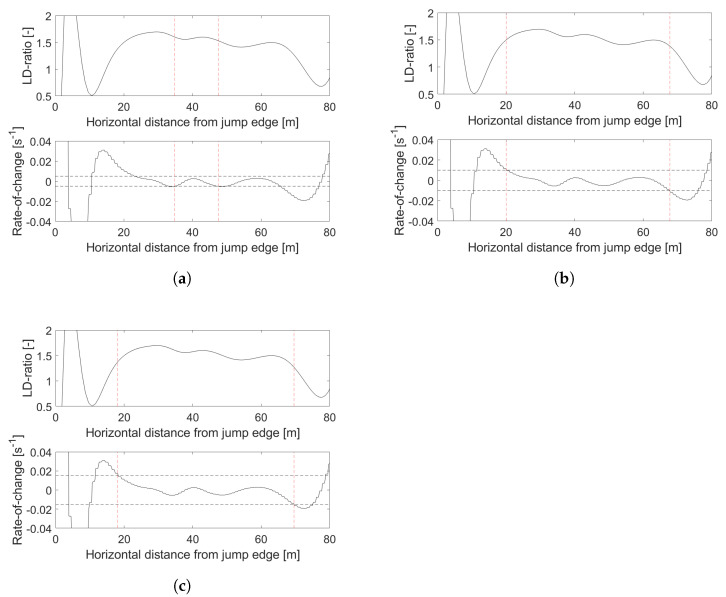
Example of a jump for which the steady glide phase was defined with the three rate-of-change thresholds (**a**) $\tau_{0}$ = 0.005 s^−1^, (**b**) τ = 0.01 s^−1^, and (**c**) τ = 0.015 s^−1^. In the lower graphs, the horizontal black dashed lines show the rate-of-change threshold and the vertical red dashed lines indicate the start and end of the steady glide phase, where rate-of-change exceeded τ.

The standard deviation in rate-of-change in the mid-region of the aerial phase p${}_{m}$ was ∼ 0.005 s^−1^; thus, $\tau_{0}$ was set to 0.005 s^−1^ and a sensitivity analysis was performed with τ ranging from 0.005 s^−1^ to 0.015 s^−1^. Figure 4 shows examples where τ was set to 0.005 s^−1^, 0.01 s^−1^, and 0.015 s^−1^ to find the start and end of the steady glide phase for one jump on HS106.

**Figure 5:**
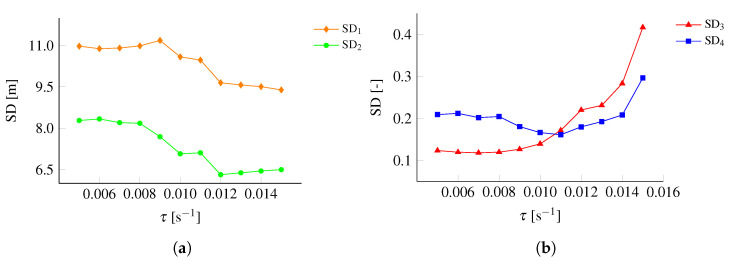
Sensitivity analysis of the standard deviation of the starting point, end point, and LD-ratio at these points for different thresholds. (**a**) displays the standard deviation of the start and end point (SD${}_{1,2}$) of the steady glide phase. (**b**) displays the standard deviation of the LD-ratio at the start and end point (SD${}_{3,4}$) for all τ tested.

As shown in Figure 4a, parts of the aerial phase where the LD-ratio is steady will not be defined as steady glide, if the τ thresholds are set too small. This effect will vary from jump-to-jump and, consequently, the standard deviation of the defined start and end point for all jumps in HS106 will be high for small threshold values and become smaller as the threshold is increased. The length of the steady glide phase will increase with increasing thresholds. If the τ threshold is too large, the algorithm will define parts of the early flight and landing preparation (Phase 3.1 and 3.1) as steady glide. In these regions, the LD-ratio is rapidly decreasing; thus, the variation in HS106 of the LD-ratio at the defined start and end of all jumps will be high. The standard deviation of the defined start and end point (SD${}_{1,2}$) and the LD-ratio (SD${}_{3,4}$) for τ between 0.005 s^−1^ and 0.015 s^−1^ of HS106 are displayed in Figure 5.

**Figure 6:**
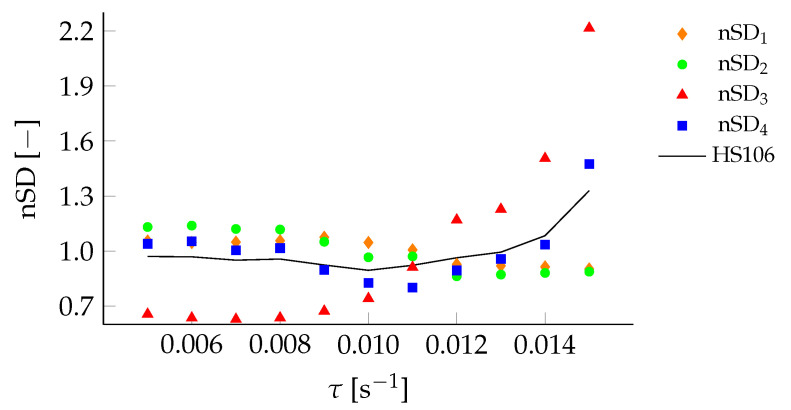
Cost function for HS106, based on nSD${}_{1-4}$ for τ in the range 0.005–0.015 s^−1^, used to find $\tau_{opt}$.

Low threshold values cause large variations in the start and end points, while high thresholds cause large variations in the LD-ratio. The cost function, based on nSD${}_{1-4.\tau }$ from the SD${}_{1-4}$ in Figure 5, is shown in Figure 6, and is used to find the optimal threshold value $\tau_{opt}$; i.e., the τ with the lowest relative standard deviation.

From this analysis, the optimal threshold value was determined to be $\tau_{opt}$ = 0.01 s^−1^.

### 3.2. Filtering Analysis

**Figure A1:**
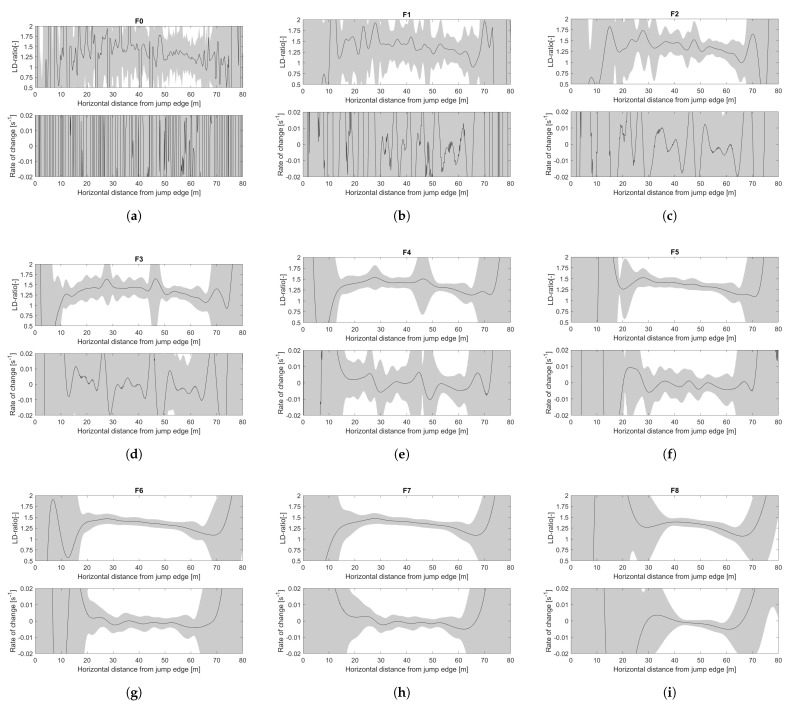

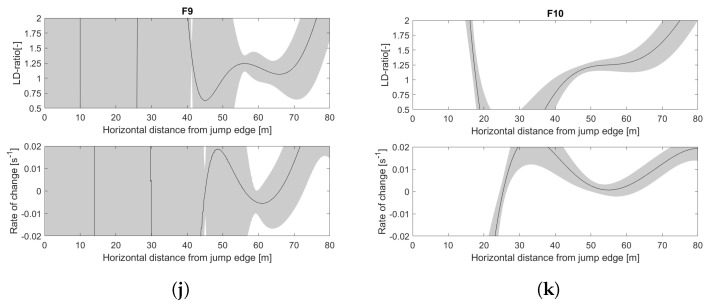
Average data from HS106 for (**a**) unfiltered data and the different filter settings in (**b**–**k**), where (**g**) displays the filter settings used by Elfmark et al. [35].

The filter settings were analyzed to highlight the effect filters have on such data. Ten different filters (F1–F10) were applied to the data, where the cut-off frequency for position, and first and second position-time derivatives varied from 10 to 0.5 Hz. The settings for F1–F10, along with the average LD-ratio and rate-of-change of the LD-ratio for all filtered and unfiltered data (F0), are shown in Appendix A. The glide phase detection algorithm was applied to all filter settings (F1–F10) and analyzed for τ between 0.005 s^−1^ and 0.015 s^−1^. After an initial check, the unfiltered data and the data from the filters F1–F4 were discarded from further analysis, as the algorithm was unable to detect any steady glide phase of ≥ 5 m in the majority of the jumps (seen in Appendix A). Filters F9 and F10 were also discarded after visual inspection (shown in Figure A1), since the signal clearly deteriorated for these filter settings; i.e., not only noise, but also substantial aspects of the signal were removed. Hence, the filters analyzed were F5–F8 and the settings for these are shown in Table 2.

**Figure 7:**
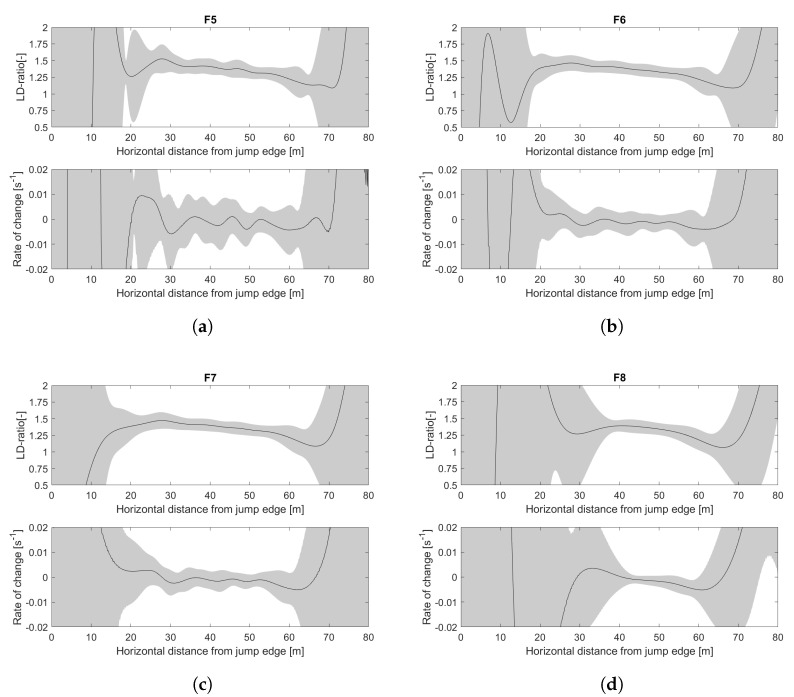
Average data from HS106 with filter setting (**a**) F5, (**b**) F6, (**c**) F7, and (**d**) F8. The upper figure shows the average LD-ratio and the lower shows the rate-of-change. Gray shaded error band displays the standard deviation with *n* = 38, and the horizontal distance from the jump edge on the *x*-axis.

Filter F6 is the setting used in the first part of this study and also in Elfmark et al. [35]. Compared to F6, F5 highlights the effect of a high cut-off frequency on the position data. The application of F7 leads to an increase in position error, while decreasing the first and second derivatives. F8 is a filter where the effect of the cut-off frequency on position data is similar to F6 with an increase in the first and second derivative filter. The average data of F6–F8 are displayed in Figure 7.

**Figure 8:**
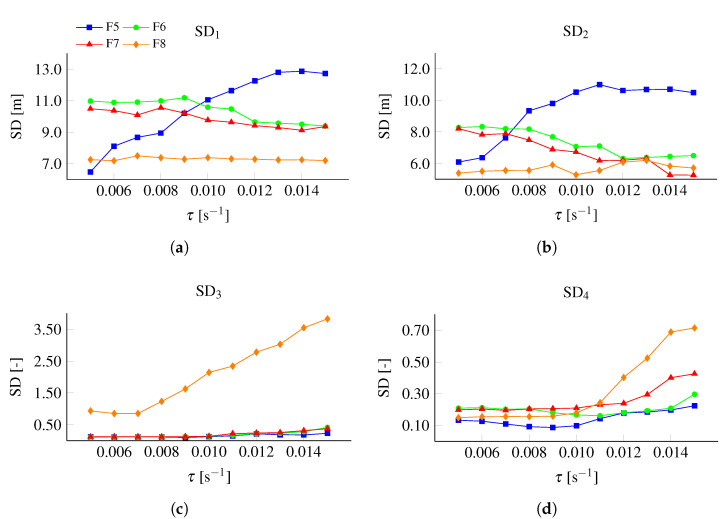
Standard deviation of defined (**a**) start point (SD${}_{1}$), (**b**) end point (SD${}_{2}$) and LD-ratio at (**c**) start (SD${}_{3}$) and (**d**) end point (SD${}_{4}$) for filter F5 (▪), F6 (•), F7 (▴), and F8 (⧫) defined by using the steady glide algorithm with τ varying from 0.005 to 0.015 s^−1^.

The effects of filter settings were analyzed by assessing SD${}_{1-4}$, when different τ were applied in the algorithm for the HS106 data, as shown in Figure 8. The variation in rate-of-change is large in F5, as shown in Figure 7a. This will cause the algorithm to not identify a steady glide phase in many jumps; i.e., start and end will be defined at p${}_{m}$, which is also shown in Appendix A (Table A2). This explains the small SD${}_{1,2}$ for small τ for F5 in Figure 8a,b. SD${}_{1,2}$ increased rapidly for increasing τ and were in total much larger than for the other filters; i.e., the data were considered as not sufficiently filtered. Filter setting F8 showed a lower SD${}_{1,2}$ than the other filters. However, SD${}_{3,4}$ was large, indicating that the algorithm had defined parts of the preparation phases as steady, i.e., the data were considered as too heavily filtered.

**Figure 9:**
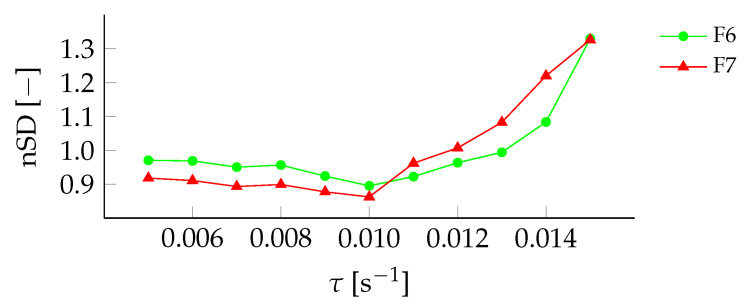
Sensitivity analysis of the cost function for different τ for filter setting F6 (•) and F7 (▴).

To investigate F6 and F7 further, the cost function of the standard deviation was analyzed to find the $\tau_{opt}$, as in Figure 6, shown in Figure 9.

Both filter settings showed similar trends with $\tau_{opt}$ = 0.01 s^−1^. Both filter settings were considered adequate for this data collection, but F6 was chosen as the average standard deviation, the aforementioned parameters were ∼2% lower on average over all thresholds, and ∼1.2% was lower at 0.01 s^−1^.

### 3.3. Hill Size and Performance Level

**Figure 10:**
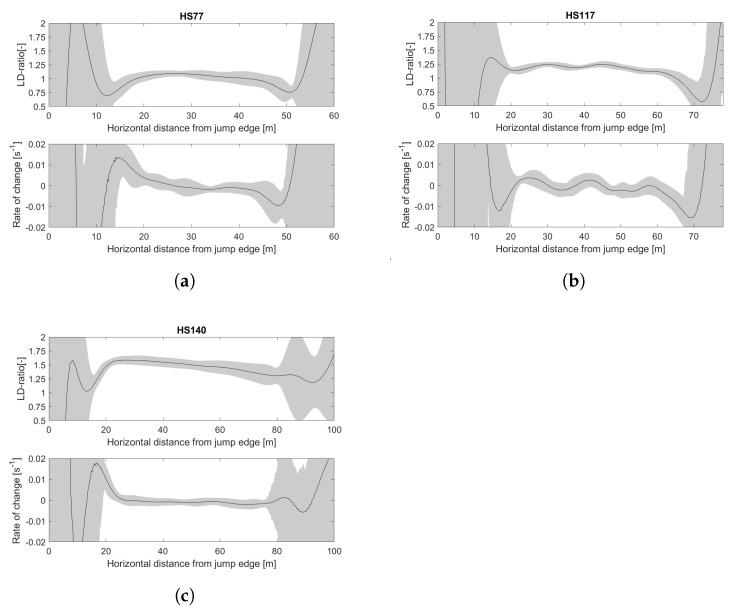
Average LD-ratio and rate-of-change in LD-ratio from the data collection in (**a**) HS77, (**b**) HS117, and (**c**) HS140.

After defining τ and filter settings, the algorithm was tested on data from other hills and ski jumpers. The jumps on HS106 and HS140 were both performed by male ski jumpers normally competing in World Cup and Continental Cup competitions and the jumps on HS117 and HS77 were performed by male and female junior athletes. Testing the algorithm on the data from hill size (HS140/HS106 and HS117/HS77) and different performance levels (HS106/HS117) aimed to assess whether the algorithm was robust in conditions and athletes other than in HS106. The average data from HS106 are displayed in Figure 3 and Figure 7 and the data from HS77, HS117, and HS140 are shown in Figure 10.

**Figure 11:**
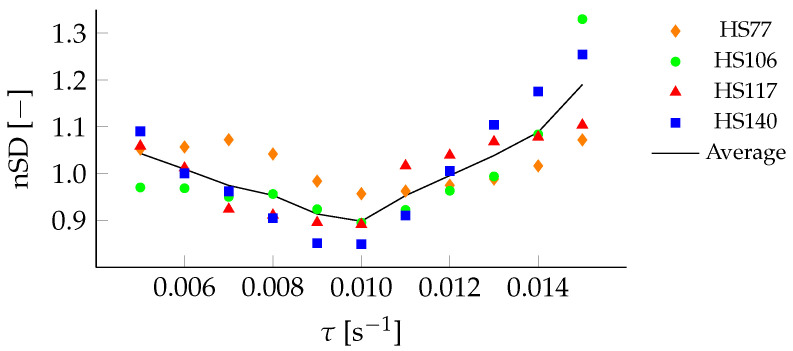
Sensitivity analysis of the cost functions for different τ at HS77 (⧫), HS106(•), HS117 (▴), and HS140 (▪), and the average across the data sets.

As was done for HS106, the data from HS77, HS117, and HS140 were analyzed with threshold values ranging from 0.005 s^−1^ to 0.015 s^−1^. The average relative standard deviation values for the different hills are shown in Figure 11 along with the average across all four hills.

The cost functions of all data sets display the same trend with $\tau_{opt}$= 0.01 s^−1^ leading to a minimal normalized SD. Hence, the properties here used to define the steady glide phase, with a maximum allowed change in the rate-of-change in LD-ratio (τ), can be used regardless of the performance level of the athletes or the hill size.

### 3.4. Possibilities and Limitations

The main aim of this study was to develop an algorithm that can identify the steady glide phase of the aerial phase in ski jumping (and, thus, also the other aerial sub-phases), independent of hill size and the performance level of the athlete. It also proposes generic definitions for the aerial sub-phases that are linked to the physical (aerodynamic) conditions, rather than to observation possible to use with sensor technology like dGNSS. We argue that this will make findings from different studies easier to compare and therefore enhance the understanding of the sport.

A methodology to find proper filter settings and the optimal rate-of-change threshold for the steady glide phase has also been suggested. Other methods than the one used here are available to measure ski jumping; for example, a video-based system, as used by Elfmark et al. [35]. The data from such systems may have different error and noise characteristics, which may require different filter settings and $\tau_{opt}$ to successfully detect the steady glide phase. Once filter settings and $\tau_{opt}$ values are established for a certain motion capture method, this study suggests that the same settings can be used across different hill sizes and performance levels of ski jumpers.

It was not an aim to investigate different types of filters in detail, but rather to highlight the importance of finding a filter that separates signal and noise in a meaningful manner and allows the steady glide phase to be detected with an approach that is objective and does not rely on subjective judgments (by observation) of ski jumpers’ actions. In this study, small ski jumping hills (HS < 77) and ski flying hills (HS >140) were not investigated. Hill sizes smaller than 77 m, however, are rarely used by athletes after the junior level. Data collection using dGNSS on hills larger than 140 m may be difficult, because of the high safety restrictions. However, the authors do not see any reason why this presented approach would be any different in ski flying. Lastly, when using dGNSS measurements, it should be noted that the antenna is mounted on the head and this is used as a point mass representation of the athlete. This is not considered a problem in this study, as the ski jumpers reach a steady posture before the start of the steady glide phase, i.e., the head position is assumed to be a good representation of the athlete for finding the steady glide phase as shown in Elfmark et al. [35], where the dGNSS head position measurement was compared to a video based system reconstructing the centre of mass.

### 3.5. Summary

This study has suggested a generic definition for the aerial phase in ski jumping. Sensor technology, such as dGNSS, makes it possible to collect precise field data from this phase, to establish a clear definition of where the steady glide phase starts and ends. This will clarify the communication of results and enable more precise research comparisons between studies in the future. In this study, the steady glide phase was defined as the period in which the rate-of-change in LD-ratio is within 0.01 s^−1^, both backward (toward take-off, 0 m) and forward from 40 m after the inrun edge. While the absolute value of the threshold (band-width) and filter settings may vary, the approach to finding the optimal rate-of-change threshold $\tau_{opt}$, may be universal among hills, ski jumpers, and motion capture methods, since the $\tau_{opt}$ was shown to be universal across different hill sizes and performance levels of ski jumpers.

## Figures and Tables

**Table 1:** Information on hill size, number of athletes, gender, performance level (WC/COC: World Cup and Continental Cup level), and number of jumps measured for the four different data collections conducted in this study.

ID	Place	Hill Size	Athletes	Gender	Perf. Level	Jumps
		(m)	(#)			(#)
HS77	Einsiedeln (CH)	77	6	Female/Male	Junior	12
HS106	Midstubakken (N)	106	8	Male	WC/COC	38
HS117	Einsiedeln (CH)	117	2	Male	Junior	10
HS140	Lillehammer (N)	140	3	Male	WC/COC	33

**Table 2:** Cut-off frequency ($f_{c}$) on position, first derivative, and second derivative for the four filters analyzed.

Filter	$f_{c}\forall$f(x,y,x)	$f_{c}\forall$f${}^{\prime }$(x,y,x)	$f_{c}\forall$f${}^{\prime \prime }$(x,y,x)
	(HZ)	(HZ)	(HZ)
F5	4	3	2
F6	2	3	2
F7	3	2	1.5
F8	2	1.5	1

## Data Availability

The data presented in this study are available on reasonable request from the corresponding author.

## Abbreviations

The following abbreviations are used in this manuscript:

dGNSS	differential Global Navigation Satellite System
F0-F10	filter settings
LD-ratio	lift-to-drag ratio
SD${}_{1-4}$	standard deviation of start (1) and end (2) of glide phase and LD-ratio at these points (3,4)
τ	rate-of-change threshold
p${}_{m}$	start of algorithm search

## Appendix A. Filter Settings

Table A1 shows the settings of all filters analyzed in the study, where the cut-off frequency for position, and the first and second derivatives varied. The average data of LD-ratio and rate-of-change in LD-ratio are displayed in Figure A1 .

**Table A1:** Cut-off frequency ($f_{c}$) for position, and the first derivative and second derivatives for all filters analyzed. F0 highlights the unfiltered data.

Filter	$f_{c}\forall$f(x,y,x)	$f_{c}\forall$f${}^{\prime }$(x,y,x)	$f_{c}\forall$f${}^{\prime \prime }$(x,y,x)
	(HZ)	(HZ)	(HZ)
F0	inf	inf	inf
F1	10	10	7
F2	8	6	4
F3	5	5	4
F4	5	3	2
F5	4	3	2
F6	2	3	2
F7	3	2	1.5
F8	2	1.5	1
F9	1	1	1
F10	0.5	0.5	0.5

Table A2 shows the percentage of the 38 jumps from HS106 in which the algorithm was able to detect a steady glide of at least 5 m for the different filter settings and τ ranging from 0.0051 to 0.015 s^−1^.

**Table A2:** Percentage of the 38 jumps from HS106 where the algorithm was able to detect a steady glide of 5 m or more for the unfiltered data F0 and the different filter settings F1–F10, with τ ranging from 0.005 to 0.015 s^−1^.

Filter	0.015	0.014	0.013	0.012	0.011	0.010	0.009	0.008	0.007	0.006	0.005
F0	0	0	0	0	0	0	0	0	0	0	0
F1	0	0	0	0	0	0	0	0	0	0	0
F2	0	0	0	0	0	0	0	0	0	0	0
F3	18.4	7.9	0	0	0	0	0	0	0	0	0
F4	94.7	84.2	81.6	81.6	78.9	78.9	73.7	52.6	44.7	28.9	18.4
F5	100	100	100	100	97.4	92.1	92.1	84.2	76.3	63.2	50
F6	100	100	100	100	100	100	100	100	100	97.4	94.7
F7	100	100	100	100	100	100	100	100	100	100	97.4
F8	100	100	100	100	100	100	100	100	100	100	100
F9	97.4	97.4	97.4	97.4	97.4	97.4	97.4	97.4	97.4	97.4	97.4
F10	100	100	100	100	100	100	100	97.4	97.4	89.5	81.6
